# Supplementary material for: Are carbon emissions trading and green financial instruments synergistic? -Comprehensive quantitative research based on content analysis
Source: PLoS One. 2024 Mar 7;19(3):e0298601. doi: 10.1371/journal.pone.0298601 (PMC10919601; doi:10.1371/journal.pone.0298601)
Supplement: S1 Data — (ZIP) [file pone.0298601.s001.zip › Data/地级市Z_F工作报告.zip!/║■▒▒/╥╦▓2╩╨/└┤╫╘╣1⁄2╓┌║┼í╛┬φ┐╦╩2╛▌═°í┐_2011.docx]

各位代表：

　　现在，我代表市人民政府向大会作工作报告，请连同《宜昌市国民经济和社会发展第十二个五年规划纲要（草案）》一并审议，并请市政协委员和列席人员提出意见。

　　一、2010年和“十一五”时期工作回顾

　　刚刚过去的2010年，在市委坚强领导下，在市人大和市政协监督支持下，我们紧紧依靠全市人民，认真践行科学发展观，面对金融危机的持续冲击，面对暴雨山洪等自然灾害的严重影响，化危为机，团结奋斗，务实拼搏，全面完成市四届人大五次会议确定的工作任务。实现生产总值1547.3亿元，增长15.8%。完成全社会固定资产投资949.5亿元，增长26.6%；全地域财政收入301.5亿元，增长27.9%；地方一般预算收入70.2亿元，增长27.9%；社会消费品零售总额550.8亿元，增长22.7%；城镇人均可支配收入14282元，增长11.2%；农民人均纯收入5980元，增长15.3%。

　　一年来，我们注重区域统筹，加快拓展发展空间。白洋工业园、生物产业园开发正式启动，宜昌高新区升格为国家级高新区；县域经济蓬勃发展，夷陵、宜都、当阳、枝江生产总值过150亿元，财政总收入过10亿元。注重培植后劲，着力推进项目建设。80个重点项目完成投资211.4亿元，三峡全通涂镀板一期、万达广场等一批重大项目相继建成。新开工招商引资项目210个。新增规模以上工业企业223家。主营业务收入过亿元企业净增68家。注重发展转型，加快调整工业结构。实现规模以上工业增加值747.5亿元，增长25.5%。装备制造业增势强劲，产值增长81.3%。欧赛电池、南玻160兆瓦硅片等一批高新技术项目竣工投产，高新技术产业实现增加值135.5亿元、增长35.7%。注重优化升级，积极发展现代服务业。第三产业实现增加值480.7亿元。平湖半岛开发等项目进展顺利，旅游业加快转型升级，接待国内外游客1522万人次、旅游收入100亿元，分别增长25.4%和27.4%。火车东站物流园、华祥商业中心等一批商贸物流项目稳步推进。注重提质增效，着力发展现代农业。实现第一产业增加值176.5亿元。农业生产全面丰收，多数产品产量创历史新高。新增市级龙头企业28家。土老憨、天峡鲟鱼商标被认定为中国驰名商标。注重改善民生，大力发展公共事业。全市财政民生投入65亿元。建成了一批文化、卫生基础设施以及廉租房。实施城区菜市场标准化改造20家。成功举办了中国屈原故里端午文化节。社会保障覆盖面扩大，城市低保标准提高。新增城镇就业9.4万人，转移农民就业8.9万人。“十二五”规划编制有序推进。其他各项工作都取得明显成绩。实现“十一五”圆满收官。“十一五”时期，是宜昌发展史上极不平凡的时期。五年来，全市人民励精图治，砥砺前行，宜昌面貌发生新的历史性变化。我们积极应对国内外发展环境的复杂变化和各种重大风险挑战，趋利避害，克难奋进，创造了“宜昌速度”，实现了弯道超越。我们举全市之力服务三峡工程，移民搬迁安置顺利完成，三峡工程全面建成、发挥综合效益，成就了中华民族百年梦想。我们深入推进“三城联创”，获得国家卫生城市、国家环保模范城市称号。五年来，全市经济建设、政治建设、文化建设、社会建设和生态文明建设都取得重大进展，谱写了宜昌改革发展的新篇章。

　　过去的五年，是宜昌综合实力跨越发展的五年。坚持打基础，抓发展，好中求快，综合经济实力显著增强。2010年，全市生产总值、全社会固定资产投资、地方一般预算收入、社会消费品零售总额分别是2005年的2.6倍、3.2倍、3.1倍和2.4倍；第一产业增加值、规模以上工业增加值、第三产业增加值分别是2005年的2.1倍、3.1倍和2.4倍；金融机构存贷款余额分别达到1923.1亿元和1426.8亿元，是2005年的3.2倍和2.9倍。五年来，开工建设投资过5000万元的项目608个，是“十五”时期的2.1倍。综合实力跃居中部地区和长江沿线同等城市发展前列，进入全国百强。

　　过去的五年，是宜昌经济结构不断优化的五年。坚持用规划引导调整，用政策激励调整，三次产业协调发展。现代特色农业体系初步形成。水果、畜牧、水产、蔬菜、茶叶、中药材六大特色产业占农业总产值比重达到78.5%。农业产业化深入推进。规模以上农产品加工企业达到427家，农民专业合作社1191家。农产品加工年产值达到584亿元。枝江市、夷陵区农产品加工年产值过百亿。夷陵区、宜都市农产品加工园成为省级示范园区。耕地保护和农业综合开发力度加大，五年落实国家、省级土地整理资金25亿元，新增耕地1.6万公顷。农业支撑保障能力提高，农机化作业综合水平达到56%，农业科技贡献率53.8%。新型工业化加速推进。化工、食品医药、电力产业不断壮大，装备制造业迅速崛起，四大支柱产业支撑作用充分显现，生物医药、新材料、电子信息、新能源、节能环保等新兴产业加快成长。五年净增规模以上工业企业729家、达到1252家。净增年主营业务收入过亿元企业183家、达到246家，过10亿元企业25家、达到30家。净增高新技术企业67家，高新技术产业产值年均增长41%。新增中国名牌产品3个、中国驰名商标5件。宜化、兴发成为全国循环经济先进企业。全面完成省政府下达的节能减排任务。第三产业突破性发展。旅游业加快转型。三峡大坝、三峡人家、清江画廊等精品景区快速发展，三斗坪、车溪等旅游名镇名村相继涌现。旅游服务功能逐步完善。

　　五年共接待国内外游客5672万人次，旅游收入374亿元，分别为“十五”时期的1.5倍和1.6倍。三峡现代物流中心建设全面推进。金东山市场、中心商务区一期等一批商业项目建成营运，净增限额以上商贸企业970家，城市商贸流通服务功能增强。金融业稳步发展，兴业、浦发、汉口银行相继在宜昌设立分支机构。

　　过去的五年，是宜昌改革开放深入推进的五年。

　　坚持深化改革，扩大开放，为科学发展提供支撑与动力。国有企业改制卓有成效，宜化、兴发、安琪等一批企业活力迸发、快速壮大。多种所有制经济竞相发展，民营经济成为市域经济强大支撑。行政审批提速，审批环节减少16%，审批时限压缩67%。农村公益性服务“以钱养事”新机制基本建立。农村集体林权制度、土地流转制度、农村金融改革逐步深化。新一轮政府机构改革全面完成。社会管理不断创新，宜昌被纳入国家社会服务管理创新综合试点。公共财政体系不断完善。医药卫生体制改革初见成效。招商引资硕果累累，五年引进产业项目864个，实际到位资金766亿元，分别为“十五”时期的1.6倍和5倍。对外开放成绩斐然，五年完成出口40亿美元，年均增长32.4%；利用外资8亿美元，年均增长14.5%。三峡库区对口支援工作成效明显，五年争取对口支援资金209亿元，经济合作项目211个。

　　过去的五年，是宜昌城乡面貌巨大变化的五年。

　　坚持以交通引领城市发展，统筹推进城乡基础设施建设。综合立体交通网络基本形成。三峡机场航空口岸顺利开通。沪渝高速、荆宜高速、江南三峡翻坝高速公路和宜万铁路建成通车。宜巴高速公路、汉宜高速铁路建设顺利。全市高速公路总里程达到347公里，铁路总里程444公里，港口年吞吐量3614万吨，85%的县市区通达高速或一级公路，95%的县乡道路达到四级以上标准，所有行政村通达沥青或水泥路。城市规模扩大，功能提升。城区建成区面积拓展到100平方公里，常住人口过100万人。新建和改造中心城区道路160多公里、城市排水排污管道110公里，沿江大道延伸段、城东大道、发展大道、临江溪污水处理厂等一批项目相继建成。城区垃圾无害化处理率达到91%，污水处理率86%。城区新增绿地530公顷，人均公园绿地面积达到10.9平方米。秭归、宜都、兴山获得“国家园林城”称号。全市城镇化率提高到47%。

　　过去的五年，是宜昌人民生活明显改善的五年。

　　坚持以人为本，统筹兼顾，促进经济社会协调发展，让发展成果惠及广大人民群众。劳动保障和就业工作力度加大。解决了一批困难家庭子女培训就业问题。五年新增城镇就业43.3万人，转移农村劳动力43.5万人，城镇登记失业率控制在4.2%以内。五项保险参保人数扩大到332.7万人次。解决了被征地农民、超龄职工、村主职干部等群体的养老保险问题，解决了“关改破”困难企业职工医疗保险、农民工工伤保险等问题。城镇职工医保政策不断完善，城镇居民医保待遇大幅提高。新型农村养老保险试点有序推进，参保人数达到35.8万人。城镇低保应保尽保，农村低保扩大到14.4万人。教育、文化、卫生等社会事业取得新进步。九年义务教育完成率达到99%，初升高比例保持在95%以上，职业教育和高等教育加快发展。人才队伍发展壮大，国管、省管专家达到188人。群众文化设施日益完善。宜昌广电中心、新宜昌图书馆、新宜昌剧院建成使用。改造、新建乡镇文化站91个，建成农家书屋1294个。《楚水巴山》荣获全国第十二届文华大奖特别奖。新闻传媒、演艺娱乐、图书音像等文化产业较快发展。广播电视实现村村通，综合覆盖率达到97%。群众文化活动丰富多彩。公共卫生服务体系加强。改造提升乡镇卫生院110家、社区卫生服务站15个，99%的村卫生室达到甲级标准。新型农村合作医疗实现全覆盖。5.8万残疾人接受康复服务。群众健身运动蓬勃开展，竞技体育水平不断提高。人口继续保持低生育水平。城镇住房保障体系逐步完善，低收入群体住房困难得到缓解。五年建设经济适用房14256套、廉租房7324套，发放租金补贴22401户，改造棚户区10570户。农村生产生活条件不断改善。落实各项惠农补贴23.3亿元。新建“一池三改”农户20.7万户，改造农村危房12912户，完成通村硬化公路10885公里，建成末级渠系3908公里。解决了99.5万人饮水安全问题。减少农村低收入和贫困人口13.3万人。新建“万村千乡市场工程”农家店1463家，村级综合服务社505家。民族地区对口扶持力度加大，落实帮扶资金9.4亿元。城乡居民家庭财富普遍增加。五年来，城镇居民人均可支配收入年均增长11.9%；农民人均纯收入年均增长14%。

　　过去的五年，是宜昌精神文明和民主法制建设不断加强的五年。坚持用邓小平理论和“三个代表”重要思想武装广大干部群众，党员干部践行科学发展观能力增强，形成了风清气正、团结实干、务实创新、科学发展的浓厚氛围。深入推进精神文明创建活动，市民素质和城市文明程度进一步提升。市政府坚持依法行政，自觉接受法律监督、工作监督、民主监督和舆论监督，认真听取人大代表、政协委员的意见和建议，五年按期办复人大代表建议1512件、政协委员提案1992件，办结率100%，解决了一批关系全局和群众关注的问题。普法依法治理工作稳步推进，广大公务员特别是领导干部带头学法用法，依法行政能力提高。政风行风建设加强。社会治安综合治理力度加大。突发公共事件应急处置能力增强。安全生产形势总体稳定。社会更加安定和谐。

　　同时，援藏援疆工作深入开展，援建汉源县项目全部完成。国防动员、民兵预备役建设和双拥工作不断加强，连续四届荣获“全国双拥模范城”称号，军政军民关系更加密切。对台、侨务、外事、宗教、保密、信访、档案、方志、妇女、儿童等各项工作都取得新的成绩，气象、防震减灾、人防、老龄等各项事业全面进步。

　　各位代表，这些成绩来之不易，是历届市委、市政府“打基础、利长远”积蓄能量释放的结果，是全市人民齐心协力、团结奋斗的结果，是社会各界大力支持、积极奉献的结果。在此，我代表市人民政府，向全市广大干部群众，向给予政府工作大力支持和有效监督的人大代表、政协委员和各民主党派、工商联、无党派人士，向在宜中省单位和广大外来投资者，向驻宜人民解放军、武警官兵，向所有关心支持宜昌建设和发展的同志们、朋友们，表示衷心感谢，并致以崇高敬意！

　　各位代表，回顾过去的工作，我们深深体会到，要把宜昌的改革、发展和建设事业推向前进，必须坚持科学发展，保持目标思路和政策措施的连续性、稳定性和协调性，始终把产业发展和项目建设摆在经济工作的首要位置；必须坚持解放思想，改革创新，用改革、开放、市场的办法解决前进中的困难和问题；必须坚持依法行政，转变政府职能，切实创造良好的经济社会发展环境；必须坚持依靠人民群众，靠团结凝聚力量，靠实干成就事业；必须坚持以人为本，把为人民谋幸福作为政府工作的出发点和落脚点。

　　我们清醒地看到，发展不够仍然是宜昌最大的实际，我们的综合实力离省域副中心城市要求还有较大差距。国际金融危机影响深远，外部环境更具复杂性和不确定性；经济增长的资源环境约束显现，经济结构优化升级任务艰巨；制约科学发展的体制机制障碍仍然存在；政府服务、治理能力和机关办事效率有待进一步提高。对这些困难和问题，我们一定勇于面对，采取有效措施，切实加以解决。

　　二、“十二五”时期发展目标与要求

　　今后五年，是宜昌大有可为的重要战略机遇期和发展黄金期。国家继续扩大内需，促进中部地区崛起，推进西部大开发和长江经济带开放开发，加大三峡库区后续发展扶持力度；东部沿海产业进一步向中西部地区转移；省委、省政府深入推进“两圈一带”战略，支持省域副中心城市加快建设；宜昌交通的区域枢纽地位逐步形成等等，将给我们带来新的发展机遇。近年来一批牵动全局、关系长远的重大基础设施和产业项目相继建成，为全市经济社会发展积蓄了能量。尤其可喜的是，全市干部群众创造了一系列服务企业、服务项目、促进发展的新机制和好办法，形成了政通人和、风清气正的人文环境，这是十分宝贵的精神财富，是我们推动发展的动力源泉。我们一定乘势而上，牢牢把握机遇和优势，积极应对挑战和风险，以更加奋发有为的精神，继往开来，努力推动全市经济转型提速，社会事业全面进步。

　　根据市委四届十二次全会精神，“十二五”时期，我们将认真践行科学发展观，顺应人民群众过上更好生活的新期待，以科学发展为主题，以转变经济发展方式为主线，以解放思想、改革开放创新为动力，全面推进经济社会发展新跨越，不断提高人民群众幸福指数，把宜昌基本建成省域副中心城市和长江中上游区域性中心城市，为全面建成小康社会和世界水电旅游名城打下具有决定性意义的基础。

　　实现上述目标，我们将牢牢把握五点基本要求。

　　一是坚定不移推进跨越发展。始终把发展作为第一要务，坚持加快发展不动摇、不折腾、不懈怠，着力保持又好又快发展的强劲态势，继续走在长江沿线和中部同等城市发展前列，下大力提升经济总量占全省的比重，下大力提升综合实力和辐射带动力，确保2015年生产总值超过3500亿元。呼应武汉、重庆两大城市圈发展，推进城市规模扩张、功能提升，加快现代化大城市建设步伐，努力把宜昌建设成区域性经济中心、交通中心、科教文化中心和三峡旅游龙头。到“十二五”期末，城区建成区面积达到160平方公里，常住人口160万人左右。

　　二是坚定不移推进转型发展。加快产业结构调整，构建以现代农业为基础、战略性新兴产业为先导、先进制造业和现代服务业为支撑的现代产业体系。加快生态文明建设，推动增长模式向资源节约型、环境友好型转变，促进速度与质量、发展与民生、增长与环境、经济与社会有机统一。

　　三是坚定不移推进协调发展。统筹城乡规划和建设，加速推进新型城镇化。重点推进城市向东延伸、跨江发展，促进中心城区、中小城市和城镇协调发展，加快形成特色鲜明、优势互补、环境优美的现代城市体系。大力发展县域经济，扎实推进新农村建设，促进城乡公共资源共建共享，构建城乡基础设施、产业布局、公共服务、劳动就业一体化格局，逐步缩小城乡差距。支持三峡库区和民族地区加快发展，逐步缩小区域差距。

　　四是坚定不移推进创新发展。大力推进思想观念、体制机制、方式方法创新，敢闯敢试、敢为人先，使创新成为发展的基本动力和干部群众的自觉行动。全面实施科教兴市和人才强市战略，建设高素质人才队伍，积极推动科技创新。着力增强自主创新能力，培育核心竞争优势，努力建设创新型城市。

　　五是坚定不移推进和谐发展。更加注重解决民生问题，更加注重发展社会事业，更加注重社会公平正义，更加注重维护公共安全，促进改革发展稳定相协调。突出就业、住房、教育、医疗、分配、社会保障等民生领域，逐步建立科学完整、有机衔接、相互配套的民生保障机制，加快提高城乡居民收入水平，提升人民群众幸福指数，促进社会和谐稳定。力争2015年，城镇居民人均可支配收入达到24000元，农民人均纯收入10000元。

　　三、2011年工作重点

　　今年是中国共产党成立90周年，也是“十二五”开局之年。开局影响全局，做好今年工作，对我们在新起点上实现发展新跨越具有非同寻常的意义。2011年全市经济社会发展主要预期是：生产总值增长13%；全社会固定资产投资增长20%，其中市县属投资增长30%；社会消费品零售总额增长18%；地方一般预算收入增长15%；外贸出口增长15%；利用外资增长10%；城镇居民人均可支配收入增长10%；农民人均纯收入增长9%；城镇登记失业率控制在4.2%以内；居民消费价格涨幅控制在4%左右；确保完成省政府下达的节能减排任务。制定这些目标，充分考虑了需要和可能，既积极进取，又切实可行。今年，我们将重点抓好以下几个方面工作。（一）强化项目主导，积极培育市场主体

　　坚持把招商引资、项目建设摆在经济工作首位，把工作着力点放在培育市场主体、增强市场活力上，努力新上一批重大项目，以项目增量推动发展转型。

　　切实加强重点项目建设。着力抓好国家、省投资项目的实施。对进入国家、省“十二五”规划的项目，加大工作力度，确保按期实施。对重大基础设施项目，加快前期工作进度，力争提前实施。认真编制三峡工程后续规划实施方案，落实配套政策，抓紧启动一批库区产业发展、生态保护和地质灾害治理项目。完善项目推进机制，解决好项目用地、能源、资金、用工等要素问题。继续抓好全通涂镀板二期和三期、三新硅业、联邦电缆、富程科技等续建项目，力争早日建成投产。尽快启动中兴汽车、丰泰油脂、安琪生物保健品、南玻硅片扩产、全通配套产业园等项目。

　　进一步抓好招商引资。加强市县招商队伍建设。不断完善招商机制和办法，推进对外宣传、项目策划等资源共享，整合招商力量，形成招商合力。组织园区招商小分队，搞好跟踪对接，增强招商实效。力争宜昌开发区东山园区全年落户项目20个以上，生物产业园落户项目20个以上，白洋和猇亭园区落户项目60个以上。加大现代服务业招商力度，吸引国内外名企名店入驻宜昌。鼓励知名企业在宜昌设立区域性总部和研发、营销中心。加强与中省企业战略合作，支持在宜中省企业做大做强。积极争取三峡对口支援项目和经济协作项目，力争到位资金40亿元。

　　加大培育市场主体力度。落实服务直通直达、并联审批、上门服务、限时办结等制度，把“依法依规、打破常规、特事特办、优质服务”的好办法、好经验转化为制度规范，内化为干部的行为习惯。深入推进中小企业成长工程，力争新增规模以上工业企业200家。加大建筑装饰业等市场主体培育力度。推进全民创业，营造非公有制经济、“草根经济”蓬勃发展的土壤和气候，让一切创造社会财富的源泉充分涌流。

　　（二）着眼产业集聚，推动各类开发区加快发展

　　加快宜昌高新区和沿江园区开放开发，是拓展宜昌城市和产业发展空间的必然选择。我们将集中力量，加大投入，创新机制，重点突破，把沿江一带建成全市经济重要增长极。

　　全力推进宜昌国家级高新区发展。积极申报出口加工、保税物流等海关特殊监管区域，提升高新区服务功能。放大高新区带动效应，统筹推进一区多园建设，全面提升东山、猇亭园区，加快发展白洋工业园、生物产业园、磁电子高科技产业园。推动东山园区转型升级，加快建成以现代服务业和高新技术产业为支撑的城市新区。加快猇亭园区三峡移民生态工业园、中小企业园建设，完善配套设施，确保供地不少于5000亩。高标准建设白洋工业园基础设施，尽快完成白云路兴建，加快白洋水厂、污水处理厂、变电站、客货运站场等建设，迅速拉开园区骨架。加快生物产业园花溪路兴建和汉宜路改造，抓紧建设供水、供电等重要配套设施。抓紧编制宜昌船舶工业园发展规划。依法有序推动开发区、园区征地拆迁，力争生物产业园征地拆迁年内基本完成，白洋工业园征地拆迁完成10平方公里。

　　创新园区投入机制。打破行政区划界限，对白洋工业园、生物产业园实行托管开发，市县两级共建共享。采取财政引导、市场运作、社会参与等多种方式，灵活运用BT、BOT等办法，拓宽园区建设投入渠道，引导更多社会资本进入园区开发，力争基础设施适度超前到位。充分利用土地、重大基础设施、国有股权等公共资产，增强融资能力，推进资产资本化。加强市城投公司、夷陵国资公司等融资平台建设，引导其积极参与园区开发。

　　推动产业向开发区、园区集聚。统筹编制城市、产业、园区发展规划，明确各类开发区、园区产业布局和功能区分，推进产业集聚，引导沿江开发区、园区和沿江城市群协调发展。正确处理城市建设与产业发展的关系，做到产业布局到哪里，城市基础设施建设就跟进到哪里，促进工业化与城镇化互融共进、相辅而行。支持各县市区开发区、园区加快发展，引领县域经济做大做强。鼓励库区县、山区县到沿江开发区、园区兴办项目，发展“飞地经济”。

　　（三）加快结构调整，着力提升工业整体竞争力

　　坚定不移走新型工业化道路，全面提高制造业能力和水平，力争规模以上工业增加值增长18%。

　　大力发展先进制造业。推动信息技术与制造业深度融合，革新生产方式，实现协同化创新、精细化生产、精准化管理，全面提升工业经济信息化水平。深入推进工业结构调整。促进化工产业逐步实现生产精细化、产业循环化，提升产品效益，力争规模以上企业今年主营业务收入突破600亿元。引导装备制造业抓住国家实施产业振兴规划机遇，走差异化发展道路，扶持龙头企业做优做强、增强产业带动力，推进生产过程智能化和生产装备数字化，提升产业核心竞争力，力争规模以上企业主营业务收入突破480亿元。支持食品加工业加强新技术研究和新产品开发，不断提高产品档次，抢占市场份额，力争规模以上企业主营业务收入突破420亿元。

　　大力发展战略性新兴产业。把发展战略性新兴产业作为转型升级、调整结构、培植可持续发展能力的重要举措，加强政策支持，营造良好环境，推动生物医药、新材料、新能源、电子信息、节能环保等产业加快发展。以建设生物产业园为契机，以安琪、人福、东阳光等企业为依托，加强与武汉国家生物产业基地、与高校科研院所的合作，着力构建技术开发、成果孵化、规模化生产相结合的发展体系，尽快把生物医药产业培育成新的支柱产业。大力支持南玻、劲森、欧赛、兴发有机硅等优强企业和项目加快发展，延伸产业链，发展配套企业，尽快把新材料、新能源、电子信息、节能环保等产业培育壮大。

　　大力发展循环经济和低碳经济。适应国家将主要污染物总量控制种类由2项增加到4项的新要求，进一步加大节能减排力度，支持循环经济、低碳经济加快发展。引导企业积极开发和应用节能技术，特别是建筑节能技术和产品。支持企业发展清洁生产，推进废物循环利用、资源综合利用，探索生态发展模式。加强公共机构和公用设施节能工作。积极推广低碳技术，培育低碳产业，倡导低碳消费。

　　抓好对企业的个性化服务。坚持“产业第一，企业家老大”理念，完善“大企业服务直通车”机制。加强生产要素协调，帮助企业缓解油电气运等突出困难。围绕企业投产、达产、稳产、销售等环节，主动帮助、协调解决企业自身无法解决的一些特殊问题。加强融资担保、人才培训、信息咨询、第三方物流等服务平台建设，为中小企业提供灵活有效的综合服务。

　　（四）立足农民增收，扎实做好“三农”工作

　　认真落实中央、省农村工作会议精神，以发展现代农业为主题，以增加农民收入为目标，进一步优化农业结构，积极推进农业科技创新，大力发展农产品加工，夯实农业基础，改善农村环境，加快建设社会主义新农村。

　　着力推进农业现代化。坚持特色发展。在巩固粮油生产的同时，进一步优化农业区域布局，加快打造柑橘、茶叶、蔬菜、畜牧、水产、食用油6个百亿产业，促进农产品提质增效。坚持标准化生产。新建改造标准果园10万亩、标准茶园7万亩。实施新一轮“菜篮子”工程，确保城市蔬菜有效供给。推广规模化、标准化、生态化养殖，不断提升养殖效益。建立健全质量安全、疫病防控体系，提高农产品质量安全水平。坚持产业化经营。突破性发展特色农产品加工业，壮大加工规模，培育产业集群，力争加工产值过百亿元的县市区达到3个，过50亿元的园区3个，农产品加工产值达到670亿元。支持稻花香集团加快实现销售收入过百亿。鼓励龙头企业开展科研攻关和技术创新，延长产业链条，争创精品名牌。引导龙头企业通过定向投入、委托生产、保护价收购、入股分红等多种形式，与农户结成利益共同体。培育多元化市场流通主体，搭建农超、农商对接平台，健全农产品集散地和销区市场网络体系，拓宽农产品销售渠道。坚持社会化服务。推行乡镇科技人员派驻制，强化农业科技信息服务，重点抓好良种、良肥、良法推广工作，建立市级农业科技示范基地10个。提高农业机械化作业水平。规范发展农民专业合作组织，力争新增合作社100家。

　　着力改善农村生产生活条件。抓住国家加大水利投入政策机遇，确保争取国家水利投资4亿元以上，完成小型水库除险加固23座，新建、改造末级渠系460公里，建设抗旱水池5000口；抓好沮漳河泵站更新改造、东风渠灌区节水改造，启动中小河流治理工程6处；治理水土流失面积80平方公里；解决10万人以上饮水安全问题。确保争取中央、省土地整理资金6亿元，改造低丘岗地15万亩、低产林8万亩，建设高产农田10万亩。加大农村道路建设投入，新建农村公路1200公里。积极推进新一轮农村电网改造升级。继续实施“十镇百村”示范工程和乡村清洁工程，完成“一池三改”生态家园建设3万户。开展区域性农村环保试点，减少面源污染。鼓励各类主体以多种形式参与小城镇基础设施建设，推动一批商贸重镇、旅游名镇加快发展。积极推进中心村建设，引导农村人口相对集中居住，建设农村新社区。落实最严格的耕地保护制度，促进土地节约集约利用。启动新一轮整村推进扶贫开发，确保搬迁扶贫4000人。大力支持五峰开展“脱贫奔小康试点县”工作。积极扶持库坝区移民生产发展，促进移民安稳致富。

　　着力拓宽农民增收渠道。整合培训资源，开展农民培训和农村实用人才开发，提高农民科技水平和转移就业能力。严格落实各项惠农强农政策，增加农民政策性收入。积极发展农村非农产业，促进农民就近转移就业，增加工资性收入。探索建立农村土地经营权、林权流转和抵押机制，增加农民财产性收入。

　　（五）围绕转型升级，大力发展现代服务业

　　把发展现代服务业作为结构调整的战略重点，积极推动服务业转型发展，规模扩张。

　　加快旅游业转型步伐。高标准编制三峡国家级旅游度假区规划，整合旅游资源，提高开发利用效益。引导区域内旅游企业联合重组、做大做强。加快推进平湖半岛、三峡游轮中心、峡口风景区等一批重点旅游项目建设。支持三峡人家、清江画廊、凤凰山屈原文化旅游区、九畹溪风景区创建5A景区，玉泉山风景区创建4A景区。继续推进旅游名镇名村建设，加强特色民居改造，加大生态走廊建设力度。不断完善城市旅游服务功能。深化区域旅游合作。创新城市形象宣传方式，扩大宣传效应，提高宜昌旅游知名度。精心办好第二届中国长江三峡国际旅游节、第六届中国宜昌长江三峡国际龙舟拉力赛、中国屈原故里端午文化节。加快建立三峡国际旅游目的地公共服务信息平台。依托丰富的人文、产业优势资源，支持研发一批工艺精湛、各具特色、附加值高的旅游商品。积极应对高铁时代来临和交通格局新变化，开展系列营销活动，开辟新的客源市场，力争全年接待游客1800万人次，旅游总收入125亿元。

　　加快推进三峡物流中心建设。全面实施三峡物流中心发展规划，进一步优化物流园区布局，加强物流基础设施建设，不断完善物流服务功能。积极推进太平溪港、茅坪港、火车东站、云池港等物流园区建设。支持仓储、货运等物流企业加快发展，积极引进国内外优强物流企业，整合物流资源和物流业务，培育大型物流企业集团。鼓励和推动工业企业主辅分离，实施物流流程再造，推动物流专业化。

　　拓展服务领域和创新服务业态。加紧筹建宜昌文化产业园，引导文化企业向园区集中。积极发展广播影视、新闻出版、数字动漫、文艺娱乐等文化产业。坚持政府引导、市场运作，打造文化旅游精品剧目，推进文化与旅游深度融合。大力发展会计审计、评估评价、法律事务、检验检测、会议展览、电子商务等生产性服务业，引导资源要素集聚，推进服务外包。积极发展体育健身、教育培训、家政服务等生活性服务业。支持流通业态创新，着力推行连锁经营、电子商务等现代商贸方式，改造提升传统商贸业。力争新增限额以上商贸企业300家。精心组织“家电下乡”、“家电以旧换新”活动。继续推进“万村千乡市场工程”和“新农村现代流通网络工程”，新建村级综合服务社240家，改善农村消费环境。合理引导住房需求，抑制投机需求，促进房地产业平稳健康发展。

　　（六）坚持扩容提质，加快推进新型城镇化

　　加强城市基础设施建设，进一步提升城市功能，强化城市管理，统筹推进新型城镇化，增强城市辐射带动力和综合竞争力。

　　逐步构建现代化城市体系。站在区域协调发展高度，强化中心城市地位和作用，抓紧完成城市总体规划修编，合理安排城市空间布局。有序推进中心城区拓展骨架，壮大规模，彰显滨江、山水、园林特色，提升城市魅力。积极支持宜都、枝江、当阳向中等城市发展，加快与中心城区融合，与“宜荆荆”城市群对接。合理确定中心镇和重点镇功能定位、产业布局、开发边界，促进城市和城镇功能互补，形成基本公共服务和基础设施一体化、网络化发展格局。积极探索农村人口转移落户城镇的政策措施。

　　加快提升综合交通功能。发挥承东启西、通南达北的节点优势，构建枢纽型综合交通运输体系。加强公路、铁路、港口、站场等基础设施建设，力争完成交通投资80亿元。扎实做好香溪长江大桥、宜湘高速公路、三峡翻坝港口等项目前期工作。加快太平溪港二期、茅坪港、云池港二期等港区建设，完善集疏运功能，着力建设亿吨组合新港，推进以港兴城。确保沪渝高速龙舟坪连接线、伍家客运枢纽站等工程年内基本完工。加快318国道万城大桥至云池段改建，加紧建设小鸦等一级路，力争陆渔路建成通车。支持保宜和宜巴高速公路、汉宜高速铁路加快建设。

　　加强城市建设和管理。继续抓好一批打基础、利长远的城市建设项目。推进东山四路、城乡路二期等城市骨架项目建设，加快城东生态新区、火车东站片区、唐家湾片区开发。取消夷陵长江大桥收费，争取庙嘴长江大桥项目立项并提前启动，抓紧实施江南大道、谭艾路改扩建，改善点军发展环境。继续开展老城区小街小巷综合治理。加强城区交通节点、停车场建设管理。继续抓好城区菜市场标准化改造。健全城市综合管理机制，强化城区网格化和数字化管理，加大综合执法力度。坚决控制和治理违章建筑。严格治理和规范户外广告。巩固“三城联创”成果，努力创建全国文明城市。

　　不断改善人居环境。加强生态文明建设，确保植树造林20万亩，提高森林覆盖率，力争建成国家森林城市。加快建设猇亭、点军垃圾处理场和污水处理厂。试行城区垃圾分类收集，改善垃圾处理系统。抓好城区沙河、合益沟治理，抓好三峡库区、清江、香溪河、黄柏河、运河等重点流域综合治理。加强饮用水水源地环境保护，实行大气污染联防联控，确保城市饮用水水源地水质达标率100%，确保城区空气质量优良天数保持在350天左右。

　　（七）突出改革创新，增强发展活力与动力

　　坚持解放思想，先行先试，大力推进重点领域和关键环节的改革，推进体制机制和科技创新，实施更加积极的开放战略，增创发展新优势。

　　深化各项体制改革。继续深化国有企业产权制度和国有资产管理体制改革，确保国有资产保值增值。支持有条件的企业实施战略性重组。进一步规范政府投资项目管理。稳步推进市政公用事业改革，完善特许经营制度，鼓励和吸引更多民间资本进入公用设施建设、社会事业等领域。完成文化体制改革和医药卫生体制改革年度任务。全面推进农村集体林权制度配套改革。稳妥推进市区财政体制改革，探索建立适应宜昌城市发展新要求的管理体制和机制。

　　增强自主创新能力。鼓励企业加大研发投入，建立研发机构，加快培育以企业为主体的区域科技创新体系。引导财政产业资金、风险投资、小额贷款、担保融资进一步向新技术、新产品研发倾斜。支持优强企业加大核心、关键技术攻关力度，开发拥有自主知识产权的技术和产品。确保新增省级以上工程技术中心2家，新增国家级高新技术企业5家。依托企业、科研单位、行业协会，加快科技创业园建设，提升孵化器功能。力争高新技术产业产值、增加值分别增长30%以上。大力实施质量兴市和商标战略。认真抓好“三峡质量奖”组织实施，引导企业推行全面质量管理。支持有实力的企业开展高新技术产业标准研制，积极参与国家和行业标准制订。确保新增中国驰名商标2件以上。

　　大力实施人才强市战略。加快人才资源开发，充分发挥人才在创新中的支撑与引领作用。加强对拔尖创新人才、急需紧缺人才、战略性后备人才的培育和引进力度，让各类人才各得其所、才尽其用。营造尊重知识、尊重人才、尊重劳动、尊重创造的良好氛围，激发人才创新活力。加大人才服务力度，解决好他们在住房、医疗、子女教育、社保等方面的实际问题。

　　提高金融创新和服务水平。进一步优化金融生态环境，积极引进股份制银行落户宜昌，积极培育小额贷款公司和村镇银行。深化农村信用社改革。鼓励金融产品创新，推广知识产权质押、动产抵押、农户大额综合授信等信贷产品。利用企业债券、中期票据、融资租赁、信托等方式，扩大融资规模。支持市城投公司发行企业债券10亿元以上。健全中小企业贷款担保体系，壮大贷款担保规模。大力发展创业风险投资。加强金融基础建设，促进银企、银政合作。利用现代化支付手段提升城乡金融服务水平。加大企业上市推进力度，力争有3家企业进入上市辅导期。加强金融监管，严厉打击非法集资等金融违法行为，维护区域金融安全。

　　加快发展开放型经济。坚持以开放促发展、促改革、促创新、促转型，不断拓展新的开放领域和空间。支持有条件的企业积极引进国外先进技术、先进装备、紧缺资源和原材料，开展消化吸收再创新，形成自主知识产权，打造自主品牌。大力培育外贸经营主体，加强化工、机电、农产品等出口基地建设，积极培育装备制造、生物医药等出口产品新优势，不断优化出口产品结构。充分利用国际国内两个市场、两种资源，积极引进外资，加快“走出去”步伐，支持有实力的企业开展境外投资，开展对外工程承包和劳务输出。加快推进水运口岸升级，完善航空口岸功能，优化“大通关”环境。

　　（八）注重民生改善，全面发展社会事业

　　把改善民生作为转变发展方式、提高发展水平的根本目的，完善公共服务，加强社会建设，不断提升人民群众幸福指数。

　　加强就业和社会保障体系建设。全面落实税费减免、小额贷款等就业扶持政策。积极开展就业援助，重点抓好高校毕业生、返乡农民工、困难群体就业。继续开展困难家庭子女志能激发、技能培训关爱活动，做好城镇“零就业家庭”就业“消零”工作。深化校企合作，开展各类职业技能培训，缓解企业用工难。实施创业带动就业工程，力争进入国家创业型城市行列。新增小额贷款担保基金2300万元，增发小额担保贷款1.8亿元，重点支持劳动密集型小企业加快发展，多渠道开发就业岗位。确保新增城镇就业7万人，帮助下岗失业人员和困难人员再就业3.5万人，转移农村劳动力7.5万人。着力推行劳动合同制度，确保城镇各类企业劳动合同签订率达到80%以上。加强劳动保障维权工作。继续扩大社会保险覆盖面，确保参保总人数达到340万人次。维护被征地农民合法权益，切实解决好他们的住房、就业和养老保险等问题。支持宜都、秭归等地推进新型农村养老保险试点。巩固和发展新型农村合作医疗制度，逐步提高筹资水平，提高住院补偿比例。开展提高农村居民重大疾病保障水平试点。努力完善社会救助体系。健全城乡低保标准动态增长机制，力争城区城市低保标准提高15%以上。完善农村低保制度，基本实现“全员纳入、应保尽保”目标。进一步加快社会化养老服务体系建设。加大保障性住房建设力度，城区新建公共租赁房1460套。扩大城区廉租房保障覆盖面，将人均住房建筑面积13平方米以下且人均月收入不足600元的困难家庭纳入廉租房租赁补贴范围。采取保障供给、加强监管、完善补贴等措施，确保物价总水平基本稳定。加强残疾人社会保障和服务体系建设。

　　加快教育、卫生、文化等社会事业发展。坚持教育优先发展战略。加大投入力度，加快发展学前教育。健全义务教育经费保障机制，完善教育网点布局规划，加强中小学校标准化建设，加强教师队伍建设，改善师资队伍结构，促进义务教育县域均衡发展。完成“校舍安全工程”建设100万平方米以上。加快宜昌职教园建设。支持三峡大学等高等院校加快发展。适应城市发展需要，科学编制规划，逐步调整城区医疗卫生资源布局。弘扬救死扶伤的人道主义精神，加强医疗卫生队伍建设管理，营造尊医重卫风尚。支持重点医院争优创名，增强区域性服务能力。落实好基本药物制度改革各项工作，切实办好乡镇、社区和村医疗卫生机构。逐步解决群众看病贵问题。做好人口与计划生育工作，促进人口均衡发展。启动新宜昌博物馆建设。加强城乡文体设施规划建设和管理，加强文化站点和文化中心户建设，丰富群众文化生活。加大对留守儿童的关爱力度，帮助留守儿童健康成长。

　　加强和创新社会服务管理。扎实开展国家社会服务管理创新综合试点，建立健全公共服务保障、公共诉求管理、重点人群管理、社会组织管理、社会矛盾化解等工作体系。完善社会应急管理机制，提高危机管理和抗风险能力。健全社会稳定风险评估和矛盾排查化解机制，把各种不稳定因素化解在萌芽状态。加大食品安全专项整治力度，加强食品药品、餐饮卫生监管，保障人民群众健康安全。加强社会治安综合治理，强化安全生产监管，确保社会和谐稳定。支持工会、共青团、妇联等人民团体参与社会管理和公共服务。加强基层民主政治建设，不断完善村民、居民自治。强化司法公正，支持审判机关、检察机关依法独立行使审判权和检察权。抓好法律援助，切实维护弱势群体合法权益。

　　同时，扎实推进国防动员工作，支持驻宜解放军和武警部队建设，不断提高预备役部队、民兵和人民防空建设水平。深入开展“双拥”创建，巩固军政军民团结。继续做好援藏援疆工作。扎实做好对口支援民族地区工作。依法管理宗教事务，注重发挥宗教界人士和信教群众在促进经济社会发展中的积极作用。加快发展老龄、防震、气象等社会事业。认真做好对台、侨务、外事、档案、方志、统计、保密等各项工作。

　　（九）加强效能建设，努力塑造人民满意政府

　　加强政府自身建设，规范行政行为，创新工作思路，提高工作效率，确保政府工作更加公正透明，更加廉洁高效，更加让人民满意。

　　坚持开拓进取，增强政府创新力。深入开展创先争优活动，弘扬勤勉敬业、奋发有为的精神，倡导勤于学习、善于研究的风气，营造勇于创新、锐意进取的氛围，切实把学习创新的成果转化为谋划科学发展的思路、领导科学发展的能力、推动科学发展的举措。

　　坚持求真务实，增强政府执行力。崇尚实干，力戒空谈，扎扎实实把政府应该做的事情做好，靠实实在在的工作业绩取信于民。深化行政审批制度改革，推进行政服务提速提效。完善目标管理办法，确保各项工作责任到位、考核到位。加强政务督查，对落实不力行为实行严格问责。

　　坚持依法行政，增强政府公信力。健全科学、民主、依法决策制度，建设法治政府。严格行政执法程序，落实执法责任，规范执法行为。加快电子政务建设，推进政务公开，切实保障人民群众知情权、参与权、表达权和监督权。依法接受人大法律监督和工作监督。主动接受政协和各民主党派、工商联民主监督。自觉接受群众监督和新闻监督。严格落实党风廉政建设责任制，推进反腐倡廉“十个全覆盖”，全面推行廉政风险防控管理，确保行政权力、公共服务公开透明运行，不断提高预防腐败工作水平。严肃查处各类违法违纪案件，切实解决损害群众利益的突出问题。

　　各位代表，回顾过去，成绩令人鼓舞；展望未来，蓝图催人奋进。让我们在中共宜昌市委的坚强领导下，在市人大、市政协的监督支持下，和全市人民一道，锐意进取，扎实工作，为加快建设省域副中心城市、实现“十二五”发展新跨越而努力奋斗！
